# *GhCKX14* responding to drought stress by modulating antioxi-dative enzyme activity in *Gossypium hirsutum* compared to *CKX* family genes

**DOI:** 10.1186/s12870-023-04419-0

**Published:** 2023-09-02

**Authors:** Tengyu Li, Kun Luo, Chenlei Wang, Lanxin Wu, Jingwen Pan, Mingyang Wang, Jinwei Liu, Yan Li, Jinbo Yao, Wei Chen, Shouhong Zhu, Yongshan Zhang

**Affiliations:** 1 State Key Laboratory of Cotton Biology, Institute of Cotton Research of the Chinese Academy of Agricultural Sciences, Anyang, 455000 Henan China; 2 National Key Laboratory of Crop Genetic Improvement, Huazhong Agricultural University, Wuhan, 430070 Hubei China; 3 The Key Laboratory for Quality Improvement of Agricultural Products of Zhejiang Province, College of Advanced Agricultural Sciences, Zhejiang Agriculture and Forestry University, Hangzhou, 311300 Zhejiang China; 4 College of Plant Science, Tarim University, Alar, 843300 Xinjiang China; 5 Zhengzhou Research Base, State Key Laboratory of Cotton Biology, School of Agricultural Sciences, Zhengzhou University, Henan, Zhengzhou, 450001 China

**Keywords:** Cytokinin oxidase/dehydrogenase, Antioxidant, Abiotic stress, Gene expression, Hormones

## Abstract

**Background:**

Cytokinin oxidase/dehydrogenase (*CKX*) plays a vital role in response to abiotic stress through modulating the antioxidant enzyme activities. Nevertheless, the biological function of the *CKX* gene family has yet to be reported in cotton.

**Result:**

In this study, a total of 27 *GhCKXs* were identified by the genome-wide investigation and distributed across 18 chromosomes. Phylogenetic tree analysis revealed that *CKX* genes were clustered into four clades, and most gene expansions originated from segmental duplications. The *CKXs* gene structure and motif analysis displayed remarkably well conserved among the four groups. Moreover, the *cis*-acting elements related to the abiotic stress, hormones, and light response were identified within the promoter regions of *GhCKXs*. Transcriptome data and RT-qPCR showed that *GhCKX* genes demonstrated higher expression levels in various tissues and were involved in cotton’s abiotic stress and phytohormone response. The protein-protein interaction network indicates that the *CKX* family probably participated in redox regulation, including oxidoreduction or ATP levels, to mediate plant growth and development. Functionally identified via virus-induced gene silencing (VIGS) found that the *GhCKX14* gene improved drought resistance by modulating the antioxidant-related activitie.

**Conclusions:**

In this study, the *CKX* gene family members were analyzed by bioinformatics, and validates the response of *GhCKX* gene to various phytohormone treatment and abiotic stresses. Our findings established the foundation of *GhCKXs* in responding to abiotic stress and *GhCKX14* in regulating drought resistance in cotton.

**Supplementary Information:**

The online version contains supplementary material available at 10.1186/s12870-023-04419-0.

## Background

Cytokinins are a class of structurally similar N6-substituted adenine derivatives with a crucial role in plant cell division, differentiation, and morphogenesis [1–4]. These processes include regulation of seed germination, leaf senescence, flower bud differentiation, floral development, fruit ripening, and all development processes throughout the whole life cycle of a plant [5]. Cytokinin oxidase/dehydrogenase (CKX) is the main enzyme that catalyzes cytokinin degradation, and it is also the only enzyme known to catalyze the inactivation by irreversible degradation of natural cytokinins in active plant cells [4, 6, 7].

Various adverse environmental conditions adversely affect the growth and development of plants, and plants have developed complex regulatory networks to cope with environmental stress during evolution [8]. Different phytohormones are involved in the regulation of plants defending against biotic and abiotic environmental stresses, then mediate the physiological and biochemical metabolic reactions to adapt to the changing environment [9, 10]. It has previously been demonstrated that several CKX family members were involved in responses to abiotic stresses in plants, such as *Arabidopsis thaliana* (*At*) [11], *Nicotiana tabacum* (*Nt*) [11, 12], Zea mays (*Zm*) [13], Oryza sativa (*Os*) [14], and *Glycine max* (*Gm*) [15]. Seven *CKX* genes isolated in *Arabidopsis* interact with CKs to participate in drought stresses [16]. The expression of *GmCKXs* and *ZmCKXs* are induced by drought and salt stress in soybean and maize [13, 15]. Meanwhile, *ZmCKX1* is a pivotal regulator in controlling the levels of active cytokinin in plant cells during maize root development [17]. Overexpression of *CKX2* leads to increased root system size and improved tolerance of transgenic tobacco plants to drought stress [11]. *SlCKXs* exert a pronounced effect on endogenous cytokinin levels changed and indirectly affected hydrogen peroxide accumulation in tomato leaf [18].

Furthermore, cytokinin oxidase/dehydrogenase (*CKX*) genes have a function in more than playing a role in defense against environmental stresses but exhibiting a broad range of essential effects in plant growth and development. It has been reported that the *AtCKX*-overexpressing transgenic tobacco plants regulated the cell cycle by decreasing the levels of endogenous cytokinins content, resulting in abnormal development of shoots and roots [19]. Meanwhile, *AtCKX1* and *AtCKX3* overexpression transgenic plants led to inflorescences reduced in the peduncle, and the capacity of floral primordium formed in apical meristem was reduced in comparison with wild type [20]. Down-regulation of *OsCKX2* responsible for the enhanced grain yield by accumulation in cytokinin of inflorescence meristem, and it was weakened the yield loss under salt stress conditions [14]. In addition, *TaCKX2.4*-RNAi and *HvCKX1*-silenced plants can be improved the yield, respectively [21, 22].

The cytokinin oxidase/dehydrogenase (*CKX*) genes regulates growth and development process may be a common feature among most higher plants. *CKX* genes are encoded by a multigene family, and the gene sequences have been identified across different species [23]. In an earlier study, cytokinin promotes fiber differentiation pre-flowering in cotton, but it inhibits fiber elongation after flowering [24]. Another study found that the *GhCKX*-silenced tobacco lines exhibited higher cytokinins content compared to wild-type plants, and *GhCKX* overexpressing inhibited the fiber growth due to the content of CKs significant decrease [25]. Recently, Xu et al. [26] have found that the down-regulation of *GhCKX3* resulted from a delay in the defoliation.

Various adverse environmental conditions adversely affect the growth and development of plants, and plants have developed complex regulatory networks to cope with environmental stress during evolution [1]. Different phytohormones are involved in the regulation of plants defending against biotic and abiotic environmental stresses, then mediate the physiological and biochemical metabolic reactions to adapt to the changing environment [2, 3]. Cytokinins are a class of structurally similar N6-substituted adenine derivatives with a crucial role in plant cell division, differentiation, and morphogenesis [4–7]. These processes include regulation of seed germination, leaf senescence, flower bud differentiation, floral development, fruit ripening, and all development processes throughout the whole life cycle of a plant [8].

Cytokinin oxidase/dehydrogenase (CKX) is the main enzyme that catalyzes cytokinin degradation, and it is also the only enzyme known to catalyze the inactivation by irreversible degradation of natural cytokinins in active plant cells [7, 9, 10]. It has previously been demonstrated that several CKX family members were involved in responses to abiotic stresses in plants, such as *Arabidopsis thaliana* (*At*) [11], *Nicotiana tabacum* (*Nt*) [11, 12], *Zea mays* (*Zm*) [13], *Oryza sativa* (*Os*) [14], and *Glycine max* (*Gm*) [15]. Seven *CKX* genes isolated in *Arabidopsis* interact with CKs to participate in drought stresses [16]. The expression of *GmCKXs* and *ZmCKXs* are induced by drought and salt stress in soybean and maize [13, 15]. Meanwhile, *ZmCKX1* is a pivotal regulator in controlling the levels of active cytokinin in plant cells during maize root development [17]. Overexpression of CKX2 leads to increased root system size and improved tolerance of transgenic tobacco plants to drought stress [11]. *SlCKXs* exert a pronounced effect on endogenous cytokinin levels changed and indirectly affected hydrogen peroxide accumulation in tomato leaf [18].

Furthermore, cytokinin oxidase/dehydrogenase (*CKX*) genes have a function in more than playing a role in defense against environmental stresses but exhibiting a broad range of essential effects in plant growth and development. It has been reported that the *AtCKX*-overexpressing transgenic tobacco plants regulated the cell cycle by decreasing the levels of endogenous cytokinins content, resulting in abnormal development of shoots and roots [19]. Meanwhile, *AtCKX1* and *AtCKX3* overexpression transgenic plants led to inflorescences reduced in the peduncle, and the capacity of floral primordium formed in apical meristem was reduced in comparison with wild type [20]. Down-regulation of *OsCKX2* responsible for the enhanced grain yield by accumulation in cytokinin of inflorescence meristem, and it was weakened the yield loss under salt stress conditions [14]. In addition, *TaCKX2.4*-RNAi and *HvCKX1*-silenced plants can be improved the yield, respectively [21, 22].

The cytokinin oxidase/dehydrogenase (*CKX*) genes regulates growth and development process may be a common feature among most higher plants. *CKX* genes are encoded by a multigene family, and the gene sequences have been identified across different species [23]. In an earlier study, cytokinin promotes fiber differentiation pre-flowering in cotton, but it inhibits fiber elongation after flowering [24]. Another study found that the *GhCKX*-silenced tobacco lines exhibited higher cytokinins content compared to wild-type plants, and *GhCKX* overexpressing inhibited the fiber growth due to the content of CKs significant decrease [25]. Recently, Xu et al. have found that the down-regulation of *GhCKX3* resulted from a delay in the defoliation [26].

The research on CKX genes in cotton is still lacking, although the *CKX* family members in model plant species have been characterized [27–30]. Previous study has identified that the *CKX* genes are induced by abiotic stresses, especially salt and drought [11–15]. Although many *CKX* gene family members from various species were investigated, the potential molecular mechanisms of the involvement of *GhCKXs* in stress responses remain unknown. Since the critical roles of CKX proteins in plant stress responses, the functions and regulatory networks of the *CKX* family in cotton were systematically investigated in this work. Our findings established the theoretical foundation for understanding the regulatory mechanism of *CKXs* in cotton under drought stress and broadening the ability of genetic diversity for stress resistance.

## Results

### Identification and phylogenetic analysis of CKXs

A BLAST search using the CKX protein sequences encoded by *ATCKX* genes as probes were performed against four sequenced cotton species (two diploid progenitors: *G. arboreum* and *G. raimondii*, and two cultivated tetraploid *G. hirsutum* and *G. barbadense*) and *O. sativa* (japonica) DNA databases as described [16, 31–35]. The protein domain was analyzed by the software of HMMER (http://www.ebi.ac.uk/Tools/hmmer/, accessed on 1 June 2022) and SMART to validate the proteins harboring the Cytokin-bind (Cytokinin dehydrogenase/FAD and cytokinin binding) domain (PF09265). A total of 101 gene sequences encoding putative members of the CKX family were identified in *A. thaliana*, *O. sativa*, *G. arboreum*, *G. raimondii*, *G. hirsutum*, and *G. barbadense*, respectively. *CKX* genes were named based on the initiation position distribution on the chromosomes.

The most widely distributed cotton species was *G. hirsutum*, widely cultivated in over 84 countries [36]. The disparity of GhCKX protein sequence lengths is slight, ranging from 429 amino acids (GhCKX13, GH_A13G2225) to 553 aa (GhCKX24, GH_D10G2783). The molecular weight (MW) of CKX proteins in *G. hirsutum* demonstrated the same trend with protein sequence lengths, which varied between 48.037 (GhCKX13) and 61.888 kDa (GhCKX24). Isoelectric point analysis was performed, and it was found that 16 GhCKX proteins were acidic (pI < 7.0, the average is 6.3), which have 11 GhCKX proteins was basic protein (pI > 7.0, the average is 8.4). The GRAVY (grand average of hydropathy) value of all CKX proteins is less than 0, suggesting that all CKX proteins are hydrophilic (Table S1).

**Fig. 1:**
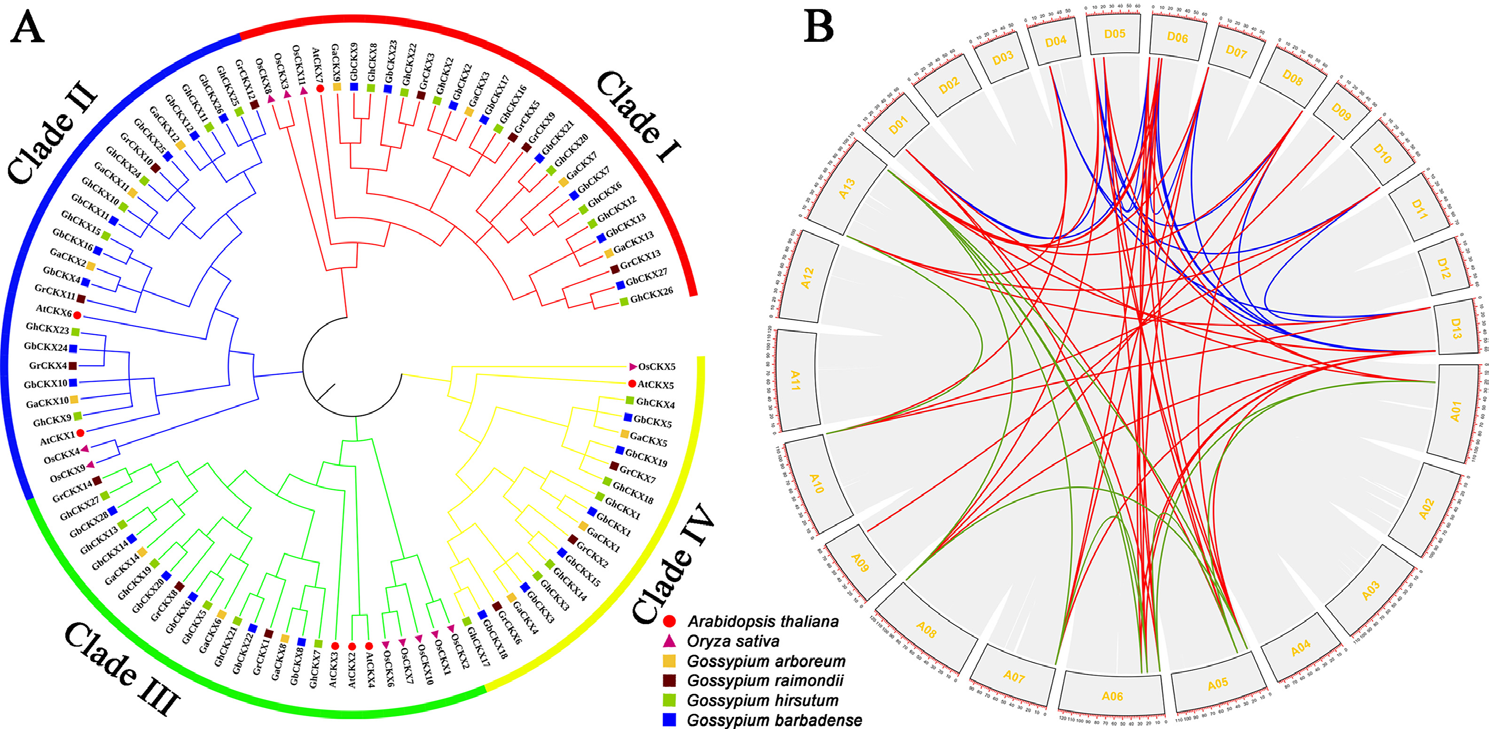
Phylogenetic tree of six species and intraspecific collinearity in upland cotton

To investigate the functional characteristics and phylogenetic evolutionary relationship of the *CKX* gene family members, the full-length sequences of the 101 proteins from *A. thaliana* (7 proteins), *O. sativa* (11 proteins), *G. arboreum* (14 proteins), *G. raimondii* (14 proteins), *G. hirsutum* (27 proteins), and *G. barbadense* (28 proteins) were examined and building a phylogenetic tree (Fig. 1A). The result shows that 101 members of the CKXs family in six organisms had evolutionary tree clustering relations and could be divided into four clades. Clade I possessed 28 members (contained 4 members from *G. arboreum*, 4 members from *G. raimondii*, 8 members from *G. hirsutum*, 8 members from *G. barbadense*, 1 members from *A. thaliana*, and 3 members from *O. sativa*), the second was Clade II (total 27 *CKX* genes, contained 4, 4, 7, 8, 2, and 2 members, respectively), followed by Clade III (total 26 *CKX* genes, contained 3, 3, 6, 6, 3, and 5 members, respectively) and Clade IV (total 20 *CKX* genes, contained 3, 3, 6, 6, 1, and 1 member, respectively). Moreover, we can infer that the *CKX* genes undergo a series of genomic amplification during evolution from diploid cotton (*G. arboreum* and *G. raimondii*) to tetraploid cotton (*G. hirsutum* and *G. barbadense*).

To identify the duplication events in *G. hirsutum*, a collinearity analysis of *CKXs* was conducted using MCScanX (Multiple Collinearity Scan toolkit) programs. Firstly, the whole genome of upland cotton was analyzed by collinear blocks analysis (Fig. 1B, gray background) and visualized by TBtools software. Meanwhile, gene duplication paralogous gene pairs *GhCKXs* were analyzed and highlighted in different colors. The green highlight indicates *GhCKXs* in A subgenome with A subgenome duplications. The blue highlight indicates *GhCKXs* in D subgenome with D subgenome duplications. The red highlight indicates *GhCKXs* in A subgenome with D subgenome duplications (Fig. 1B). This could be deduced from the *CKX* family members’ amplification in upland cotton largely derived from the chromosomal segment duplicates (Table S2).

The green lines indicates At-At internal duplications. The blue lines indicates Dt-Dt internal duplications. The red lines indicates At-Dt duplications.

### Phylogenetic classification, gene structure, and conserved motif analyses of *GhCKXs*

**Fig. 2:**
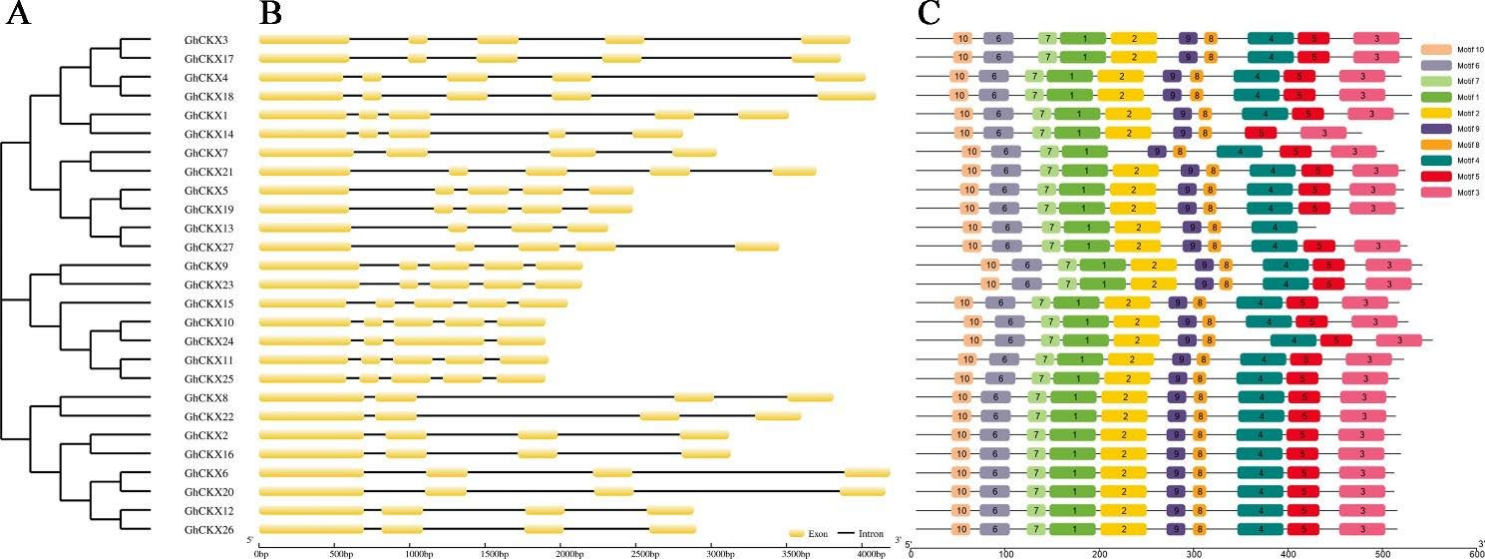
The phylogenetic tree gene structure and conserved motifs of GhCKX sequences in *G. hirsutum*. **A** the phylogenetic tree of GhCKX protein sequences in *G. hirsutum* (bootstrap neighbor-joining method); **B** the exon-intron structure of *GhCKX* genes. Black line, intron; yellow box, coding sequence (CDS). **C** Conserved motifs of GhCKX proteins (maximum motif set to 10)

Phylogenetic classification, gene structure, and conserved motif analyses of *GhCKXs* could provide a significant reference for *CKX* genes evolution analysis. The 27 *GhCKXs* were divided into 4 clades, and all four clades exhibit a highly conserved distribution of exon and intron features (Fig. 2A). The number of introns in Clade I-IV is 4, 4–5, 4–5, and 5, respectively (Fig. 2B). The conserved motifs of *CKX* genes were identified using MEME software (http://meme-suite.org/tools/meme, accessed on 1 June 2022); a total of ten significantly conserved motifs were analyzed and named motif1 to motif10, and the schematic distribution of 10 conserved motifs among the CKX proteins as shown in Fig. 2C. The overall structure of the CKX protein family is very conserved, all *CKX* homologs contained motif1 to motif10 except for GhCKX14 (lacked motif4), GhCKX7 (lacked motif2), and GhCKX13 (lacked motif3 and motif5). Of those, the distribution of motifs was identical in Clade I and Clade II, motif1 and motif6-10 exist in all GhCKX proteins. This result indicated that *CKX* genes were highly conserved during the evolutionary process and might play a similar functional role in plant development and defense.

### Chromosomal localization and collinearity analysis gene duplication analysis of Gossypium *CKXs*

**Fig. 3:**
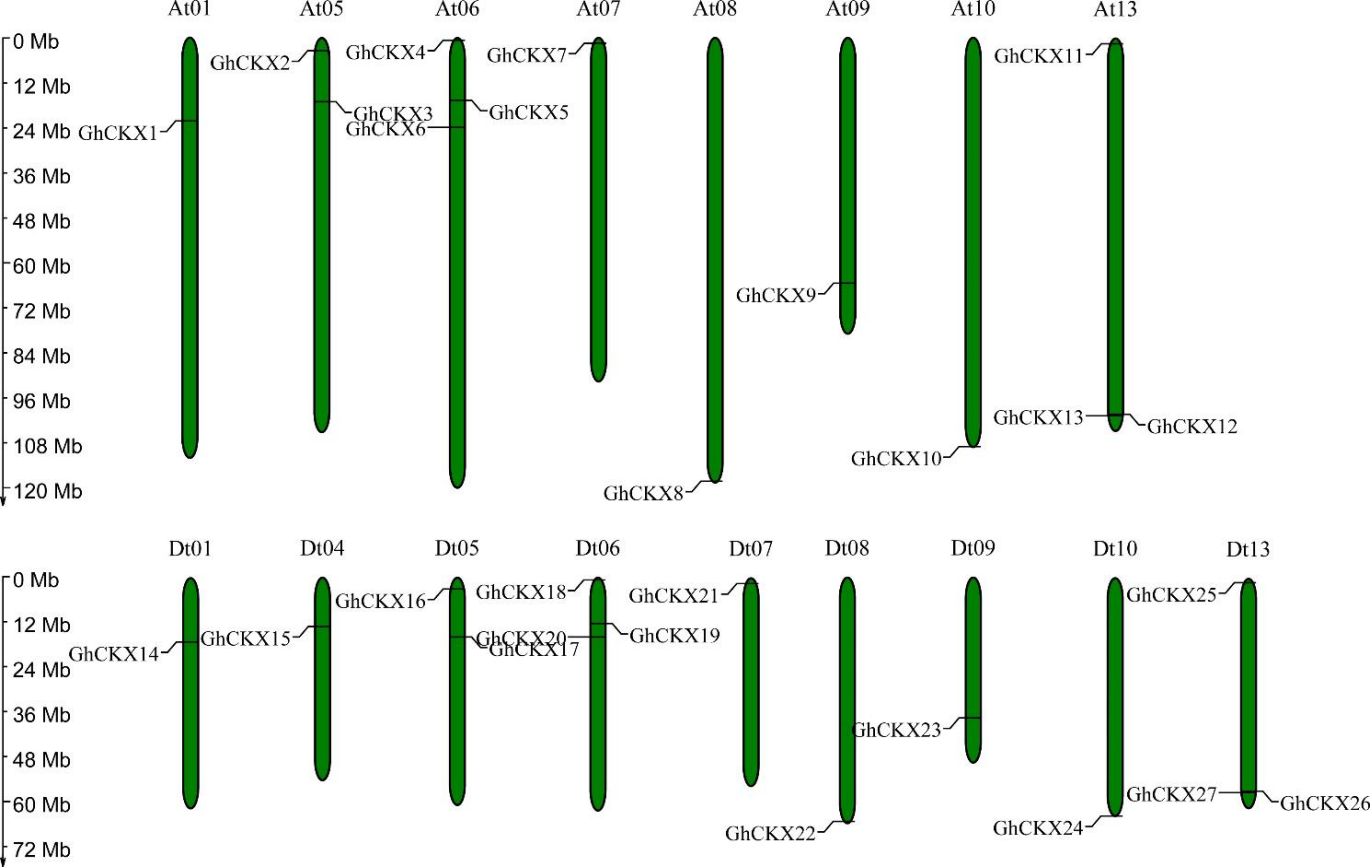
Chromosomal positions of *CKXs* from upland cotton with gene-id shown on the left and right side. The scale bar on the left side represents the position of the chromosome length

Genome-wide identification of *CKX* gene family members was performed in upland cotton, and a total of 27 *CKXs* were identified in the genome of upland cotton (TM-1). These *GhCKX* genes were renamed from *GhCKX1* to *GhCKX27* according to their distribution on the chromosomes. *GhCKXs* were unevenly distributed in 17 of the 26 chromosomes of *G. hirsutum*, excluded At02, At03, At04, At11, At12, Dt02, Dt03, Dt11, and Dt12 (Fig. 3). Chromosomes At06, At13, Dt06, and Dt13 had the highest number of *CKX* genes (3 members). For the remaining chromosomes, only one or two *CKX* genes were presented.

### Analysis of *cis*-elements in the promoter of *GhCKXs*

**Fig. 4:**
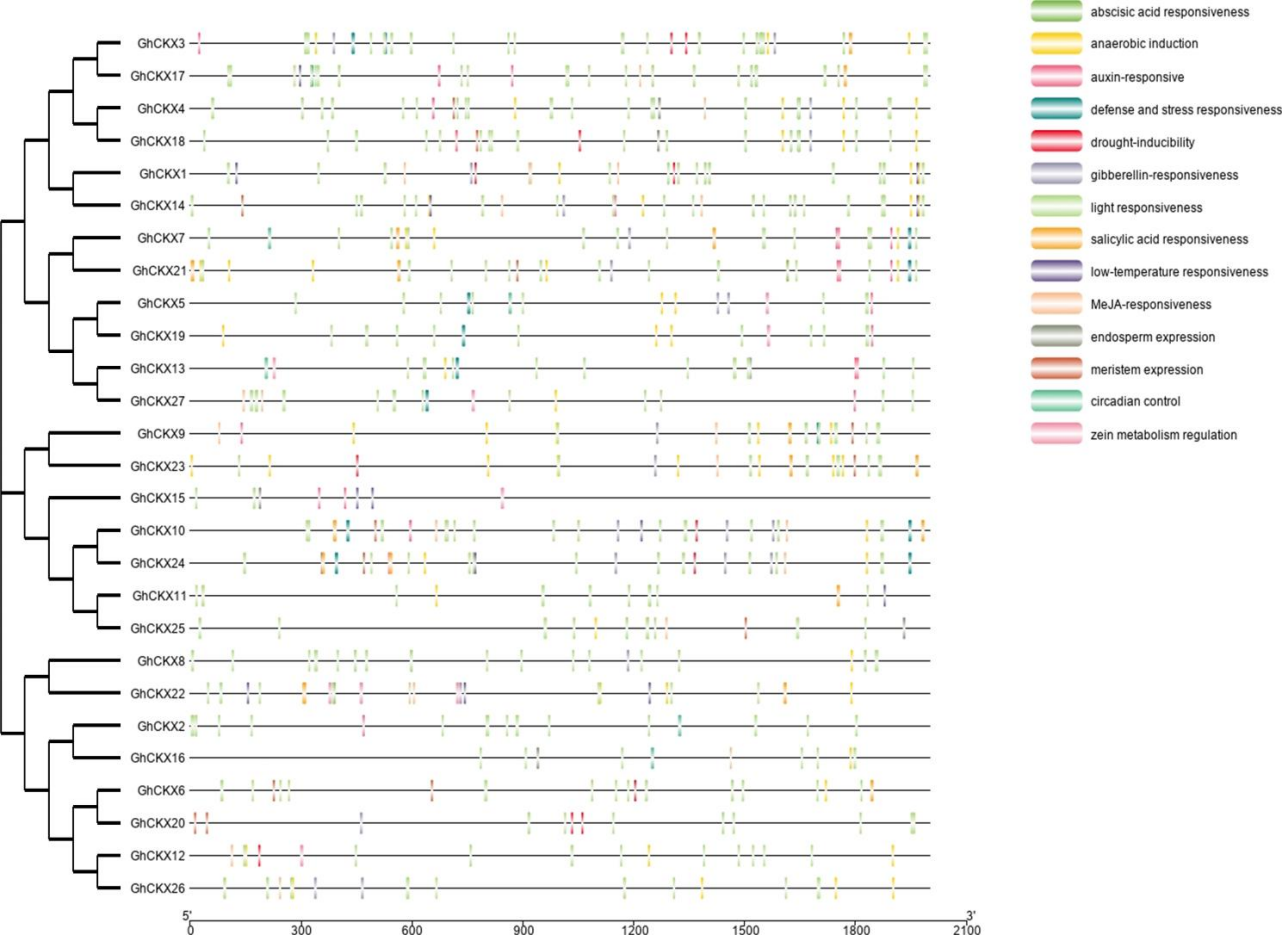
Analysis of *cis*-acting elements in the upstream promoter region of *GhCKXs*. Differently colored boxes represent unique identified *cis*-elements

Promoter regions typically contain a number of *cis*-acting elements and regulate gene expression. For *cis*-acting element prediction, PlantCARE used the upstream sequences of 27 *GhCKX* genes (2000 bp upstream from the initiation codon). Based on the analysis of the *cis*-acting elements in the promoters of the *GhCKX* family members, we found that most of the promoters contain a number of regulatory elements related to hormones and stress responses (Fig. 4). Among these elements, TATA-box and CAAT-box have the highest number of the basal regulatory element. Four categories, such as plant hormone response, defense and stress response, light response, and tissue-specific *cis*-acting elements, were identified and classified. Among plant hormone response elements, auxin-responsive (TGA and AuxRR-core), gibberellin-responsive (TATC-box, P-box, and GARE-motif), salicylic acid-responsive (TCA), abscisic acid-responsive (ABRE), and MeJA-responsive (CGTCA-motif and TGACG-motif) were predicted. The defense and stress response elements, such as defense and stress-responsive (TC-rich repeats), low-temperature responsive (LTR), anaerobic induction (ARE and GC-motif), and drought response elements (MBS) were predicted. In addition, it also includes a *cis*-acting regulatory element related to light response (ACE, G-box, Sp1, GT1-motif, MRE, Box4, CAG-motif, GA-motif, I-box, and AE-box) and tissue-specific *cis*-acting elements (endosperm expression, GCN4_motif; meristem expression, CAT-box; zein metabolism regulation, O2-site; and circadian). There are several *cis*-acting elements found in each *GhCKX* gene’s promoter region, which indicates that the *GhCKX* gene family members may play an important role in plant growth and developmental processes.

### Expression profiling of *GhCKX* genes

#### Expression patterns of *GhCKXs* under different tissues and multiple stresses

**Fig. 5:**
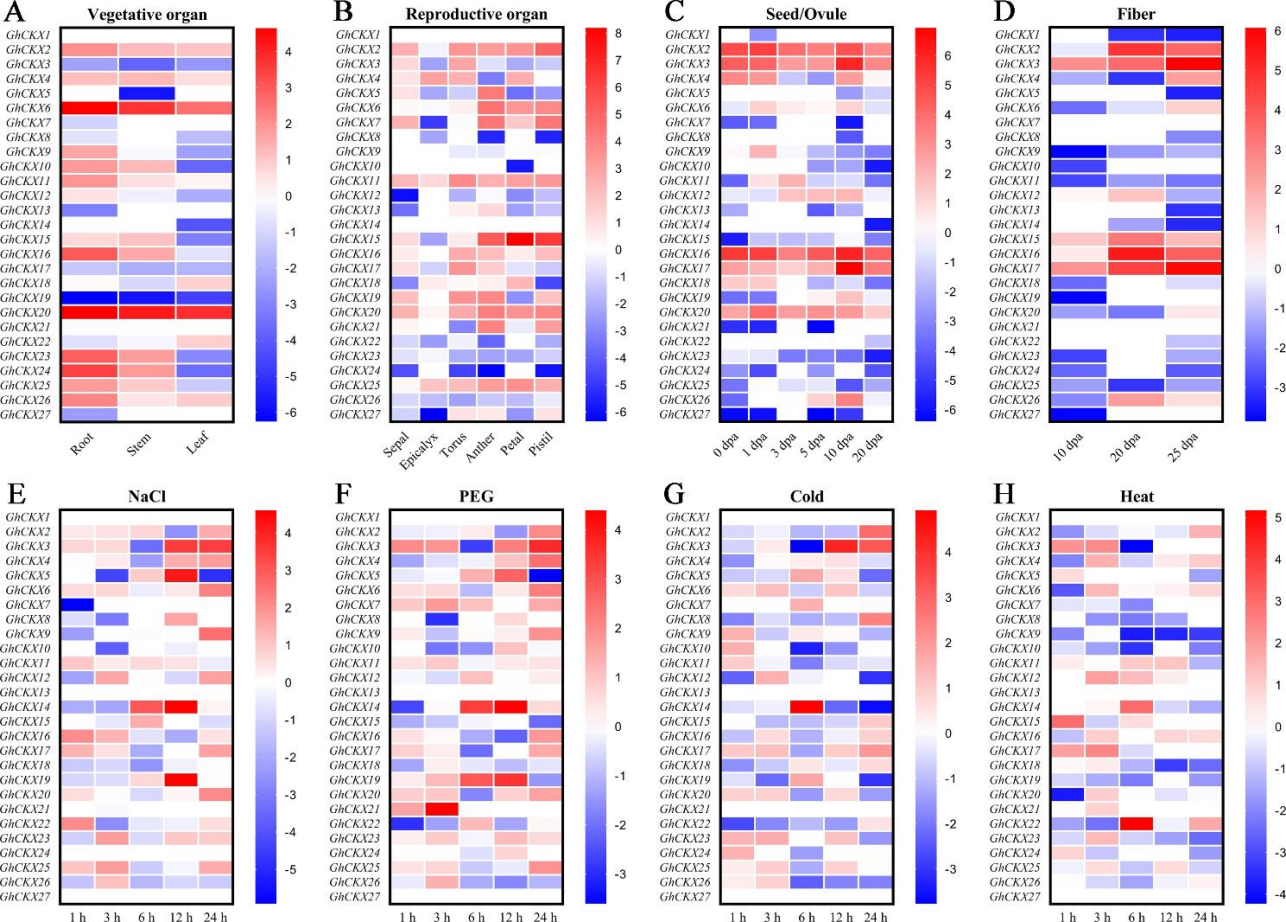
Expression patterns of *GhCKX* genes in multiple tissues (**A-D**, vegetative organ, reproductive organ, seed/ovule, and fiber) and different abiotic stress treatments (**E-H**, salt, drought, low and high temperatures) of upland cotton. The red in the legend represents high expression levels, and the blue indicates low expression levels. The expression heat map was generated based on logarithms of the FPKM values

The expression patterns of *GhCKXs* were analyzed in different tissues and various abiotic stress of *G. hirsutum* in order to better investigate the potential roles in the processes of cotton growth and development. The FPKM values of public transcriptome data among the eleven organs/stages and four abiotic stress treatments were normalized and visualized by plotting the heatmap (Fig. 5). The expression trend of *GhCKXs* in different tissues indicated the genes of *GhCKX6* and *GhCKX 20* were highly expressed in vegetative organs, especially in the root (Fig. 5A). On the other hand, only *GhCKX15* was highly expressed in reproductive organs (petal and pistil, Fig. 5B). *GhCKX2*, *GhCKX3*, *GhCKX16*, and *GhCKX17* were highly expressed in different ovule and fiber stages (Fig. 5C and D). *GhCKX3* almost possessed high expression levels in different abiotic stress (Fig. 5E-H). The expression levels of *GhCKX5*, *GhCKX14*, and *GhCKX19* reach the peak after 12 h of NaCl and PEG treatment (Fig. 5E-H). The expression level of *GhCKX14* reaches the peak after 6 h 4℃ treatment too, this suggests *GhCKX14* might be essential in responding to different abiotic stresses (Fig. 5E-H). Besides, *GhCKX21* exhibited a higher expression level after 3 h PEG treatment, and *GhCKX22* shown expression level highly after 6 h 37℃ treatment (Fig. 5E-H). These results demonstrate that the *GhCKX* gene family members play an indispensable role in cotton’s development and stress responses.

### Validation of the expression of *GhCKX* genes under abiotic stress and phytohormone treated using qRT-PCR

**Fig. 6:**
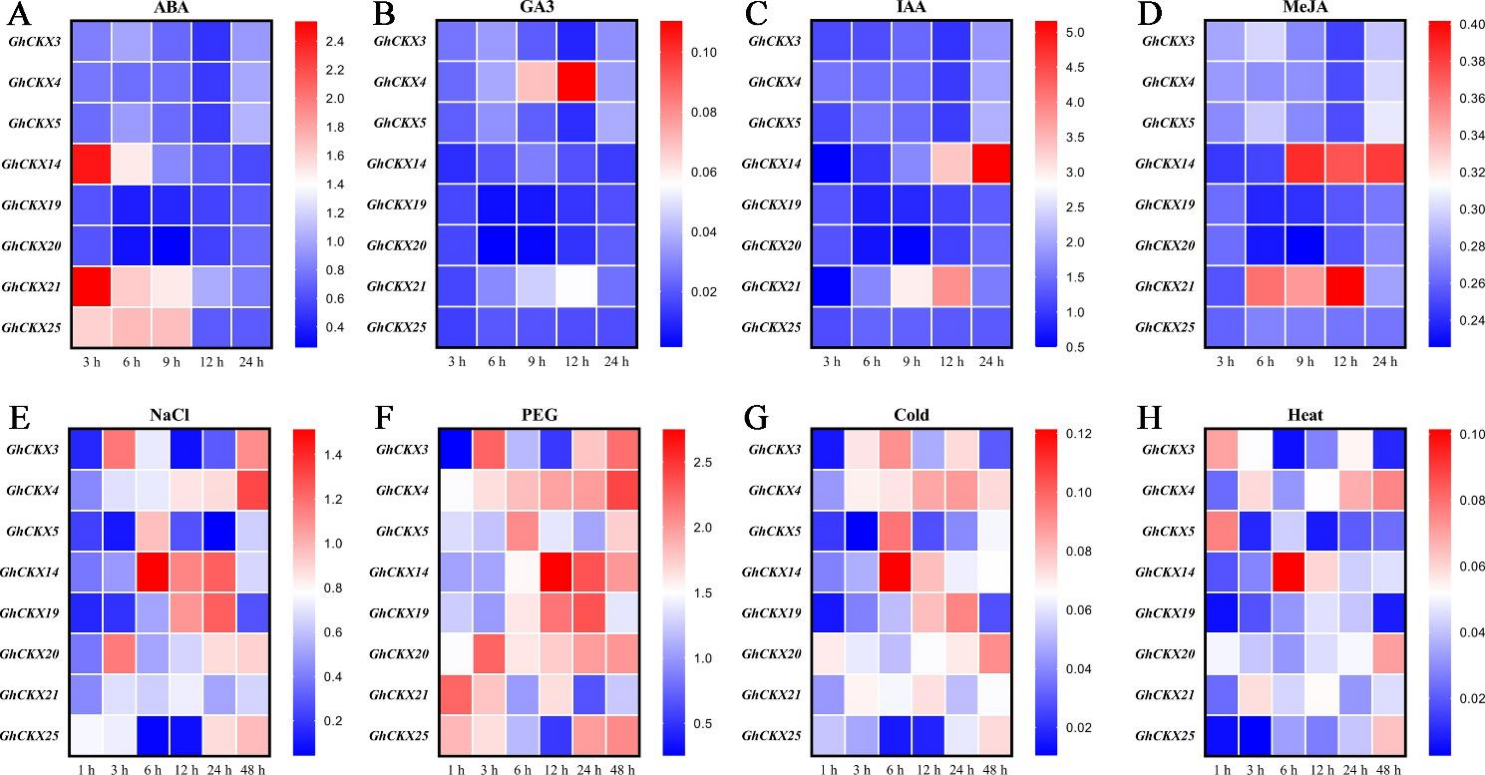
Expression profiles of *GhCKX* genes expression under various phytohormone treatments (**A-D**, ABA, GA_3_, IAA, and MeJA) and stress conditions (**E-H**, salt, drought, low and high temperatures) at different time points. The red in the legend represents high expression levels, and the blue indicates low expression levels. The expression heat map was generated based on the relative expression by 2^−∆∆CT^ method

To investigate the pattern of *GhCKX* gene expression under various hormones (ABA, GA_3_, IAA, and MeJA) and abiotic stress (salt, drought, cold, and heat) treatment, eight *GhCKXs* were chosen for the qRT-PCR. *GhCKX14* showed considerable expression under ABA, IAA, MeJA, salt, drought, cold, and heat conditions at certain time points (Fig. 6). Under GA_3_ treatment, only *GhCKX4* was up-regulated at 9 and 12 h compared to control (water blank). *GhCKX25* showed only traces of expression detected under ABA treatment. *GhCKX21* was abundantly expressed in ABA, IAA, MeJA, and drought treatment, and it was lower expressed in GA_3_, salt, cold, and heat treatment. Additionally, expression of *GhCKX3* was almost absent under hormone treatment, but it quickly responded to abiotic stresses. Similarly, the expression pattern of *GhCKX5*, *GhCKX19*, and *GhCKX20* were consistently distinct from *GhCKX3* expression under various hormones treatment. *GhCKX5* showed the highest peak of expression at 6 h under salt, drought, and cold treatment. *GhCKX19* exhibited the highest peak of expression at 24 h under salt, drought, and cold treatment. *GhCKX20* exhibited a higher expression level at 3 h under salt and drought treatment. These results illustrate that *CKX* gene family members could play a vital role in mitigating the harmful impact of abiotic stress conditions in cotton, and *GhCKX14* might be a key gene essential for stress-responsive.

### Gene interaction network

**Fig. 7:**
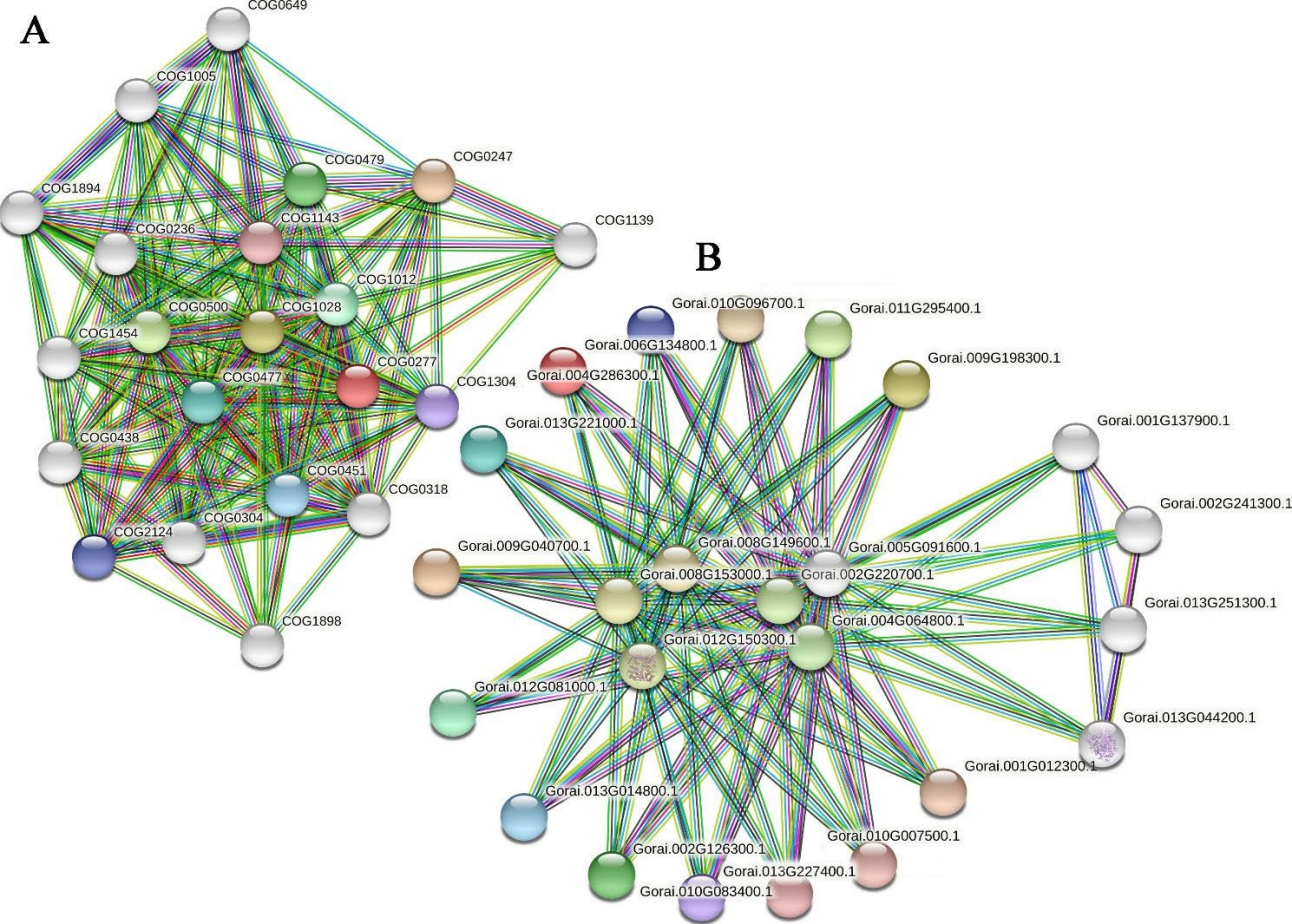
Interaction network of CKX proteins. **A** Interaction network of CKX proteins families. **B** Interaction network of GrCKX proteins with other proteins. The light green lines represent the protein-protein interaction based on textmining; dark green lines represent the protein-protein interaction based on gene neighborhood; black lines represent the protein-protein interaction based on co-expression; blue lines represent the protein-protein interaction based on gene co-occurrence; purple line represent the protein-protein interaction based on protein homology

The Database of Search Tool for the Retrieval of Interaction Gene/Proteins (STRING v11.5) was used to construct the protein-protein interaction (PPI) network. To explore the functional relationship between the CKX proteins, the PPI network was predicted using a search of protein families’ names (Fig. 7A) and multiple sequences (Fig. 7B). Based on protein family searching, protein-protein interaction between the CKX protein family (COG0277: FAD/FMN-containing dehydrogenase) and other predicted functional partners were identified (Fig. 7A). According to the score, the top ten functional partners are Fe-S oxidoreductase (COG0247), NAD(P)-dependent dehydrogenase, short-chain alcohol dehydrogenase family (COG1028), SAM-dependent methyltransferase (COG0500), Succinate dehydrogenase/fumarate reductase (COG0479), Acyl-CoA reductase or other NAD-dependent aldehyde dehydrogenase (COG1012), MFS family permease (COG0477), Nucleoside-diphosphate-sugar epimerase (COG0451), Cytochrome P450 (COG2124), FMN-dependent dehydrogenase (COG1304), and Formate hydrogenlyase subunit 6/NADH (COG1143), respectively. By the method of multiple sequences search using homologous genes in *G. raimondii*, a total of 14 *GhCKX* genes were identified interacting with others (Fig. 7B). These proteins are 4-hydroxybenzoate polyprenyltransferase (Gorai.008G149600.1 and Gorai.008G153000.1), Presequence protease (Gorai.012G150300.1), Nitrate reductase [nadh]-like (Gorai.002G220700.1, Gorai.004G064800.1, Gorai.005G091600.1, and Gorai.013G044200.1), Sulfite reductase (Gorai.001G137900.1 and Gorai.013G251300.1), respectively. Besides, the proteins of Monodehydroascorbate reductase (Gorai.002G241300.1) were also predicted. All these results indicate the CKX family probably involved in redox regulation included oxidoreduction or ATP levels to mediate plant growth and development; it also performed the predicted items of the protein-protein interaction network.

### Silencing of *GhCKX14* compromises cotton resistance to drought stress

**Fig. 8:**
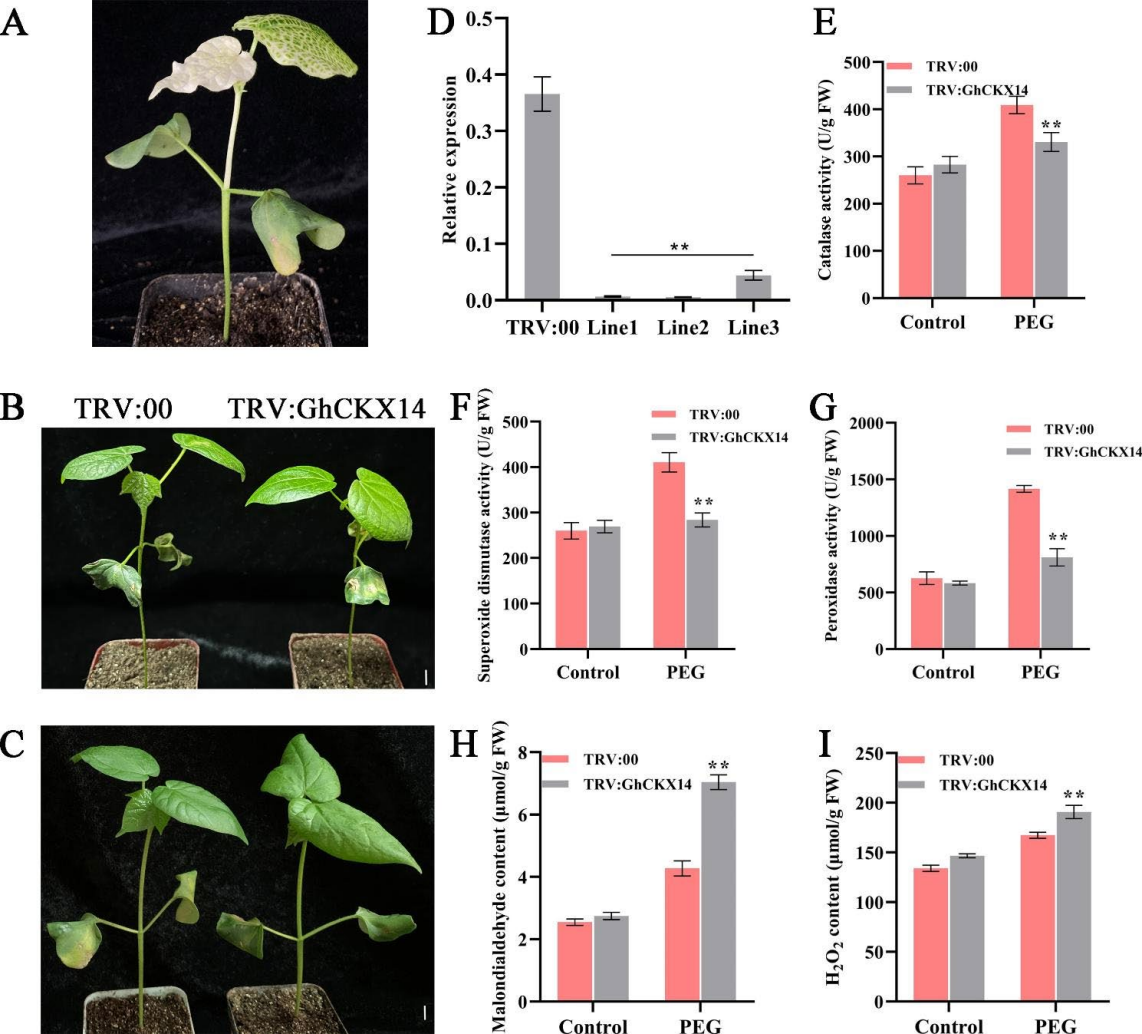
Silencing of *GhCKX14* gene reduced the tolerance of cotton plants to drought stress. **A** Plant with the albino phenotype (TRV:GhCLA1, positive control). **B** Phenotype of the negative control (TRV:00) and transgenic plants with *GhCKX14* gene silenced (TRV:GhCKX14) under normal condition. **C** Phenotype of the negative control (TRV:00) and transgenic plants with *GhCKX14* gene silenced (TRV:GhCKX14) under drought condition for 48 h. **D** Relative expression of *GhCKX14* in the control plants (TRV:00) and silenced plants (TRV:GhCKX14). *GhActin* gene was used as an internal control. **E-I** Physiological indicators were measured in plants cultivated in control and drought conditions. **E** The catalase (CAT) activity; **F** The superoxide dismutases (SOD) activity; **G** The peroxidase (POD) activity; **H** The malondialdehyde (MDA) content; (**I**) H_2_O_2_ content; Error bars indicate the standard deviation estimated by three independent experiments

The *GhCKX14* gene demonstrated a trend of sustained elevation in response to PEG treatment within 12 h according to RNA-Seq. In order to investigate the potential role of the *GhCKX14* gene under drought stress in upland cotton, the functional analysis was performed using the VIGS method. Ten days after inoculation, the positive control plants (TRV:CLA) began to display an albino phenotype, which suggests the genes were silenced (Fig. 8A). Two weeks after infection, the plants of TRV:00 and TRV:GhCKX14 were subjected to drought or salt stress. Under drought conditions, the TRV:GhCKX14 plants exhibited the crinkling phenotype in leaves compared with control plants (Fig. 8B and C). Quantitative real-time PCR (qRT-PCR) was performed to validate the expression level of negative control (TRV:00) and silenced plants (TRV:GhCKX). As shown in Fig. 8D, the expression level of silenced plants was significantly decreased compared with the negative control.

To study the effects of *GhCKX14* response to drought stress, the changes in physiological metrics contained MDA, H_2_O_2_, and antioxidant content, including POD, SOD, and CAT were measured after plants were treated with 20% PEG6000 for 12 h (Fig. 8C). After drought stress treatments, the antioxidase activities (CAT, SOD, and POD) in the TRV:GhCKX14 plants were significantly higher than TRV:00 plants (Fig. 8E, F, and G), but the contents of MDA and H_2_O_2_ in the silenced plants were significantly decreased compared with those in the negative control plants (Fig. 8H and I). These results suggest that silencing of the *GhCKX14* gene could reduce the physiological performance tolerance of cotton plants to drought stress.

## Discussion

The cytokinin oxidase/dehydrogenase (*CKX*) gene family is widely characterized in land plants, participating in biological processes of plant growth and development, abiotic stress, and hormone responsive [37]. Previous studies have identified that 7 *CKX* family members in *Arabidopsis thaliana* [20], 11 members in rice (*Oryza sativa*) [38], 17 members in soybean (*Glycine max*) [39], 23 members in oilseed rape (*Brassica napus*) [40], and 9 members in *Medicago truncatula* [41]. In the present study, 14, 14, 27, and 28 CKXs were recognized in *Ga* (*G. arboreum*), *Gr* (*G. raimondii*), *Gh* (*G. hirsutum*), and *Gb* (*G. barbadense*), respectively. Allotetraploid cotton *G. hirsutum* (AADD) probably experienced two whole genome duplications (WGD) during evolution and originates from the hybridization of two diploid ancestors (*G. arboreum* and *G. raimondii*) [33, 42]. The number of *CKX* gene family members in *Gh* was less than the sum of the number of these two diploids, suggesting the gene loss occurs after the hybridization of two diploid ancestors (Fig. 1A). In parallel, the activities of *GhCKXs* replication might play an essential role during gene evolution. A similar phenomenon of genomic replication events emerged in the case of rice [43], bread wheat [44], barley [45], and maize [46]. The evolutionary tree displayed that *CKXs* from cotton and other plant species, including *G. arboreum*, *G. raimondii*, *G. hirsutum*, *G. barbadense*, *Arabidopsis thaliana*, and *Oryza sativa* were categorized into four main groups (Fig. 1A). Remarkably, the exon-intron organization of *CKX* genes in each group was not comparabe, and they all presented the four or five (Fig. 2), suggesting these genes have a possibility to involve in the matching roles associated with multiple abiotic stress-related signals. We found that most *GhCKX* genes contain all these ten motifs and showed conserved domains of flavin adenine dinucleotide (FAD)-binding and cytokinin-binding among the 27 *GhCKX* genes (Fig. 2). Especially in Clade I and Clade II, a high degree of conservation is displayed. These indicate the *CKX* gene family is evolutionarily conserved and play similar molecular functional roles.

To illustrate the important roles of *CKX* genes in regulating abiotic stress responses, the analysis of *cis*-acting elements in promoter regions was carried out (Fig. 4). In the current study, we have predicted the type of stress response elements (drought response) and hormone response elements (auxin-responsive, MeJA-responsive, gibberellin-responsive, salicylic acid-responsive elements, and abscisic acid-responsive) were consistent with prior studies in maize [47], rice [48], and oilseed rape [40].

The gene spatio-temporal expression patterns provide important clues for investigating gene functions [49]. Analysis of *CKX* gene expression patterns across various tissues found that *GhCKX6* and *GhCKX20* were highly expressed in the root, which suggests that *GhCKXs* are probably a response to abiotic stress by modulating the development of the root system. This finding is in accordance with previous studies on the knockout of the *HvCKX1/3* gene in barley [50]. *GhCKX15* was highly expressed in petals and pistils, suggesting it could play the roles during gynoecium development. And apart from that, the high expression of *GhCKX2/3/16/17* during ovule and fiber development was in agreement with previous studies in upland cotton [51]. Additionally, downregulation of *GhCKX3b* significantly increased cytokinin contents to enhanc seed yield and fiber yield of cotton found in a recent study [52].

Based on the transcriptome data, the heat maps of expression profiling of *GhCKXs* under various stresses were constructed. Like *GhCKX14* and *GhCKX19* showed higher expression against salt and drought stress. Similarly, *GhCKX14* exhibited higher expression in response to extreme temperatures (4℃ and 37℃), and *GhCKX4* showed increased expression under various stresses. Most of the *GhCKX* genes showed higher expression in at least one of the four stresses, in particular under PEG-induced drought stress. Validation of qRT-PCR shown that *GhCKX14* and *GhCKX21* can provide responses to ABA, IAA, and MeJA signals, *GhCKX4* can provide responses to gibberellin signals and *GhCKX25* rapid responses to ABA signals. These results correspond to stress- and phytohormone-responsive elements of promoter element prediction. Results of expression patterns from RNA-Seq data are also in accordance with qRT-PCR-based relative expression for 8 selected *GhCKX* genes (Figs. 5 and 7).

Reactive oxygen species (ROS) in vivo will be produced and increased when plants are exposed to abiotic stresses; a cascade of physiological and biochemical reactions ensues to mount an effective stress response, including facilitating the activities of antioxidative enzymes [53]. To avoid or mitigate injuries caused by abiotic stresses, plants have developed an efficient enzymatic antioxidant defensive system, SOD, CAT, and POD play a major protective role. In addition, the contents of H_2_O_2_ and MDA are closely related to abiotic stress tolerance. After silencing gene *GhCKX14* with TRV-VIGS, we found the silenced cotton plants exhibited a phenotype of drought-sensitive and affected leaf development. Subsequently, the results of physiological indicators displayed a significant decrease in the SOD, CAT, and POD enzyme activity in silenced plants leaf compared to the negative control. Meanwhile, the contents of MDA and H_2_O_2_ were more accumulated. These results coincided with the previous research [54, 55]. Based on more credible and detailed evidence, it is demonstrated that beyond the TRV:GhCKX14, other CKX proteins may also have the ability to regulate drought tolerance due to the conserved FAD-binding and cytokinin-binding domain. Besides, GhCKXs were found to be involved in redox regulation, including oxidoreduction or ATP levels in protein-protein network analysis. It is in quite consistent with our results about diversified expression patterns in hormone and stress response of GhCKXs and their subcellular localizations, including vacuolar and extracellular. It has been shown previously that *CKXs* improve the tolerance of environmental stress [11, 40, 56]. In the current study, these discoveries provided more supporting evidence that *CKX* genes play an essential role in defending against abiotic and hormone stresses via modulating antioxidant enzyme activity across diverse plant species.

## Conclusions

In the present study, a total of 13, 14, 27, and 28 *CKX* genes were identified in four species of the *Gossypium* genome. To provide in-depth insight into the *CKX* gene family evolution in cotton genome,

phylogenetic relationship and synteny, gene structure, conserved motifs, *cis*-elements, protein-protein interaction network, and spatiotemporal-specific expression patterns within the *G. hirsutum* were systematically investigated. Public transcriptome data showed that some *GhCKX* genes might play significant roles in ovules and fibers development and response to abiotic stresses. Validation of results with qRT-PCR against different hormones and abiotic stress treatments indicated that several *GhCKX* genes probably contribute to stress responses and hormone signaling pathways. Current research reveals the role of the *GhCKX14* gene in cotton drought resistance by modulating the antioxidant enzyme activities. These results will lay the foundation for elucidating the roles of *GhCKX* genes in cotton developmental processes and response to various stresses and hormones.

## Materials and methods

### Plant material, growth conditions, and treatments

TM-1 (upland cotton) was chosen for this experiment and was collected from the Institute of Cotton Research Chinese Academy of Agricultural Sciences (CAAS) germplasm resources preservation library. After the seeds were delinted by sulfuric acid, healthy and plump seeds were selected and planted in the field environments to sample the root, stem, leaf, pollen, sepal, anther, ovules (0, 1, 3, 5, 10, and 20 days post anthesis, DPA), and fibers (10, 20, and 25 DPA). TM-1 seeds were planted in an artificial climate chamber at the cycle of 16 h light (12,000 Lx, 28 ℃) and 8 h dark (0 Lx, 25 ℃). Salt (200 mM NaCl), drought (20% PEG6000), cold (4 ℃), and heat (37 ℃) treatment was applied at the third true leaf unfolding stage, and ultrapure water was used as a negative control. The sample was collected from top-one leaves after 1, 3, 6, 12, 24, and 48 h of abiotic stress treatments. ABA (abscisic acid, 100 mM), auxin (indole-3-acetic acid, 20 mM), GA_3_ (gibberellins, 50 mg/ml), and MeJA (methyl jasmonate, 100 mM) were sprayed onto the leaves and treated with various hormones for 3, 6, 9, 12, and 24 h were collected. Every five treated leaves were randomly collected and stored at -80 °C until subsequent RNA extraction.

### Identification and phylogenetic analysis of CKX protein

AtCKX protein sequences were downloaded from the Arabidopsis Information Resource database (TAIR, https://www.arabidopsis.org/, accessed on 1 June 2022), and searched using Blast with the CKX gene sequence in *G. arboreum* (CRI) [31], *G. raimondii* (JGI) [32], *G. hirsutum* (ZJU) [33] and *G. barbadense* (ZJU) [33] genome. All the cotton genome sequences were downloaded from the Cottongen database (https://www.cottongen.org/, accessed on 1 June 2022) [34]. *O. sativa* (japonica) genome information was downloaded from the NCBI database. After removal of the redundant sequences, the results obtained were validated using SMART (http://smart.embl-heidelberg.de/, accessed on 1 June 2022) [35] and Pfam (http://pfam.xfam.org/, accessed on 1 June 2022) [57] databases and protein domain identification. Based on the above-identified results, we obtained protein and DNA sequences for all *CKX* genes from *A. thaliana*, *O. sativa*, and four *Gossypium* species. The physicochemical properties including of the constituent amino acids (molecular weight, charge, isoelectric point, and grand average of hydropathy) of identified CKX protein sequence, were conducted with programs available at the ExPASy Proteomics Server (http://web.expasy.org/protparam, accessed on 1 June 2022) [58]. The subcellular localization was predicted using the Soft Berry online website (http://linux1.softberry.com/berry.phtml, accessed on 1 June 2022). CKX protein sequences were aligned by the ClustalW program [59], and phylogenetic analysis was performed using a neighbor-joining (NJ, bootstrap with 1000 replicates) tree by MEGA7.0 software [60].

### Collinearity and chromosomal locations of *CKXs*

Analysis of gene duplication within the species along with collinearity using MCScanX (Multiple collinear scanning toolkits) [61], and schematic drawn by TBtools (https://github.com/CJ-Chen/TBtools, accessed on 1 June 2022). The information about CKX genes’ location on chromosomes was visualized using MG2C (Map Gene 2 Chromosome, v2.0, http://mg2c.iask.in/mg2c_v2.0/, accessed on 1 June 2022).

### Analysis of the gene structure and conserved protein motifs

Gene structural diagram was performed using the online gene prediction tool GSDS v2.0 (http://gsds.cbi.pku.edu.cn/, accessed on 1 June 2022) [62]. The conserved motifs were analyzed using the online software MEME (http://meme-suite.org/tools/meme, accessed on 1 June 2022); the maximum number of motifs was set at 10. The sequence logo information of the motif is shown in Figure S1.

### Analysis of *cis*-elements in the promoter regions of *GhCKXs*

Based on the selected promoter sequences − 2000 bp from the transcription initiation site of *GhCKXs*, we performed the *cis*-element analysis using the PlantCare website (http://bioinformatics.psb.ugent.be/webtools/plantcare/html/, accessed on 1 June 2022) [63]. The sequences of 2000 bp in upstream regions of *CKXs* were downloaded from CottonFGD [64]. Specific information regarding *cis*-acting elements presented in Table S3, and the statistics of *cis*-acting elements are in Fig S2.

### Virus-induced gene silencing of *GhCKX14* in upland cotton

Constructing the knockdown vectors tobacco rattle virus (TRV)-based virus-induced gene silencing (VIGS) system was used to silence *GhCKX14* as previously described [65]. The silenced fragment of *GhCKX14* was designed by the web-based SGN VIGS Tool (https://vigs.solgenomics.net/?tdsourcetag=s_pcqq_aiomsg, accessed on 5 May 2022) and cloned into the pTRV2 vector by using specific primers (Table S4). The vector of TRV:CLA (Cloroplastos alterados) as a positive control and the vector of TRV:00 as a negative control. The recombinant plasmid TRV:GhCKX14 was transferred into *Agrobacterium tumefaciens* GV3101. The plasmid TRV1 (subsidiary vector), TRV:00, and TRV:CLA were transferred into GV3101 at the same time. Bacterial liquid were collected and resuspended in infiltration buffer (10 mM MES, 10mM MgCl_2_, and 200µM acetosyringone), taking cells to an OD600 reached 0.8–1.2. Subsequently, equal volumes of subsidiary vector (TRV1) with negative control (TRV:00), positive control (TRV:CLA), and silenced gene (TRV:GhCKX14) cultures were mixed. The solution was placed at room temperature for 3 h protected from light before infiltration into the abaxial side of the cotyledons of cotton seedlings. The plants were placed 24 h at dark after the injection, then placed in an artificial climate chamber at the cycle of 16 h light (12,000 Lx, 25 ℃) and 8 h dark (0 Lx, 23 ℃). When plants of the positive controls showed albino phenotype, suggesting that the genes have been silenced, and the expression levels of *GhCKX14* in TRV:00 and TRV:GhCKX14 were detected with real-time PCR. Plants were grown to 24 days old and treated with 20% PEG6000 solution for the indicated time, and ultrapure water was used as the control. Three biological replicates and three technical replicates were set up, and each experimental group contained 10 plants at least. The *GhCKX14* gene-specific primers sequences for VIGS are presented in Table S4.

### Expression analysis of *GhCKXs*

The RNA-seq data of *G. hirsutum* acc. TM-1 was downloaded from NCBI to analyze the expression pattern of *GhCKXs* under different tissues, stages, and abiotic stress (salt, drought, cold and heat) [33]. The heat-map of *GhCKXs* expression pattern was drawn using TBtools software by Fragments Per Kilobase of exon model per Million mapped fragments (FPKM) values (Table S5).

The leaves of TRV:00 and TRV:GhCKX14 plants were reselceted and put in liquid nitrogen rapidly, and the total RNA was extracted by FastPure® Plant Total RNA Isolation Kit (RC411-C1, Vazyme, Nanjing, China). Subsequently, the first cDNA strand was generated by a reverse transcription kit of Vazyme (R212). Real-time PCR was performed with HiScript II QRT SuperMix for qPCR (+ gDNA wiper, R223, Vazyme, Nanjing) using the CFX96 RT-PCR detection system (Bio-Rad) and *GhUBQ7* as the internal control. All the gene-specific primers sequences are presented in Table S6. Results of qRT-PCR were analyzed using a method of 2^−ΔΔCT^ [66]. All experiments were set with three biological replicates and three technical replicates, and the output results were analyzed using the student’s t-test.

### Gene interaction network of the CKX proteins

STRING online website (https://string-db.org/, accessed on 1 June 2022) was performed to predict the interaction network CKX proteins with the minimum required interaction score as 0.400, and max number of interactors to show no more than 10 interactors. The interaction network predicts based on active interaction sources: textmining, experiments, databases, co-expression, neighborhood, gene fusion, and co-occurrence.

### Determination of the related-antioxidants physiological parameters

To investigate the function of *GhCKX14* in response to drought stress, the TRV:00, TRV:GhCKX14, and mock plants were soaked in 20% PEG6000 solution at the trefoil stage. Fifteen replicates were performed for drought and water conditions. After the leaves were harvested from silenced plants and negative control, the malondialdehyde (MDA) content, hydrogen peroxide (H_2_O_2_) content, catalase (CAT) activities, superoxide dismutase (SOD), and peroxidase (POD) were determined using the MDA (BC0025), H_2_O_2_ (BC3595), CAT (BC0205), SOD (BC0175), and POD (BC0095) assay kit (Solaibao Science & Technology Co., Ltd, Beijing, China) as previously described [67–70].

### Electronic supplementary material

Below is the link to the electronic supplementary material.

Supplementary Material 1

Supplementary Material 2

## Data Availability

The datasets generated and/or analyzed in this study are available in the CottonFGD website (https://cottonfgd.net/), RNA-Seq data downloaded from NCBI (https://www.ncbi.nlm.nih.gov/) and the accession number was PRJNA490626. AtCKX protein sequences were downloaded from the TAIR database (https://www.arabidopsis.org/servlets/Search?type=general&search_action=detail&method=1&show_obsolete=F&name=CKX&sub_type=gene&SEARCH_EXACT=4&SEARCH_CONTAINS=1). The data used in the study is publicly available.

## Abbreviations

RT-qPCR: Quantitative real-time polymerase chain reaction
ATP: Adenosine triphosphate
VIGS: Virus-induced Gene Silencing
Ga: *Gossypium arboreum*
Gh: *Gossypium hirsutum*
Gr: *Gossypium raimondii*
Gb: *Gossypium barbadense*
CKX: Cytokinin oxidase/dehydrogenase
At: *Arabidopsis thaliana*
Nt: *Nicotiana tabacum*
Zm: *Zea mays*
Os: *Oryza sativa*
Gm: *Glycine max*
MW: molecular weight
AVY: grand average of hydropathy
MCScanX: Multiple Collinearity Scan toolkit
TCA: Tricarboxylic Acid Cycle
ABRE: Abscisic acid-responsive element
LTR: low-temperature responsive
ARE: AU-rich element
FPKM: Fragments Per Kilobase of exon model per Million mapped fragments
PEG: Polyethylene glycol
ABA: Abscisic Acid
GA3: Gibberellin A3
IAA: 3-Indoleacetic acid
MeJA: Methyl Jasmonate
PPI: Protein-protein interaction
NADH: Nicotinamide adenine dinucleotide
NAD: Nicotinamide adenine dinucleotide
TRV: Tobaccorattlevirus
H2O2: Hydrogen peroxide
MDA: Malondialdehyde
CAT: catalase
SOD: superoxide dismutase
POD: superoxide dismutase
WGD: whole genome duplications
FAD: flavin adenine dinucleotide
ROS: Reactive oxygen species
CAAS: Chinese Academy of Agricultural Sciences
DPA: days post anthesis
NCBI: National Center for Biotechnology Information
