# Supplementary material for: GhCKX14 responding to drought stress by modulating antioxi-dative enzyme activity in Gossypium hirsutum compared to CKX family genes
Source: BMC Plant Biol. 2023 Sep 2;23:409. doi: 10.1186/s12870-023-04419-0 (PMC10474641; doi:10.1186/s12870-023-04419-0)

# *GhCKX14* responding to drought stress by modulating antioxi-dative enzyme activity in *Gossypium hirsutum* compared to *CKX* family genes.

Tengyu Li[1,2†], Kun Luo[3†], Chenlei Wang[1], Lanxin Wu[1], Jingwen Pan[4], Mingyang Wang[5], Jinwei Liu[4], Yan Li[1], Jinbo Yao[1], Wei Chen[1], Shouhong Zhu[1*], Yongshan Zhang[1,2,4,5*]

[1] State Key Laboratory of Cotton Biology, Institute of Cotton Research of the Chinese Academy of Agricultural Sciences, Anyang 455000, Henan, China.
[2] National Key Laboratory of Crop Genetic Improvement, Huazhong Agricultural University, Wuhan 430070, China.
[3] The Key Laboratory for Quality Improvement of Agricultural Products of Zhejiang Province, College of Advanced Agricultural Sciences, Zhejiang Agriculture and Forestry University, Hangzhou 311300, China.
[4] College of Plant Science, Tarim University, Alar 843300, Xinjiang, China.
[5] Zhengzhou Research Base, State Key Laboratory of Cotton Biology, School of Agricultural Sciences, Zhengzhou University, Henan, Zhengzhou 450001, China.

## Supplementary Figure

**Supplementary Fig S1. Specific sequence logo of motif**
**Supplementary Fig S2. Statistics of *cis*-acting elements**

**Fig S1. Specific sequence logo of motif**

**motif 1**

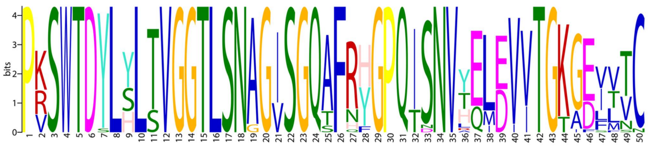

**motif 2**

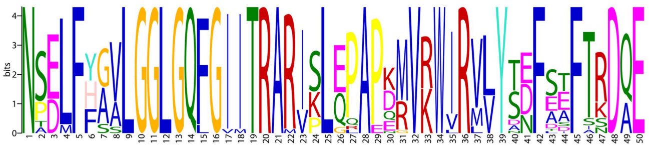

**motif 3**

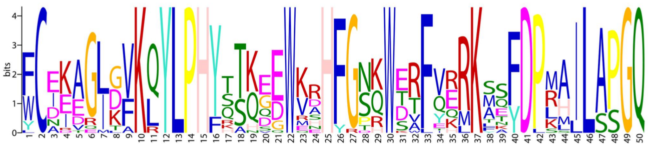

**motif 4**

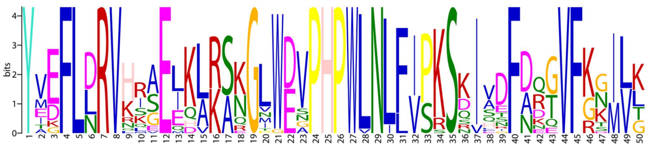

**motif 5**

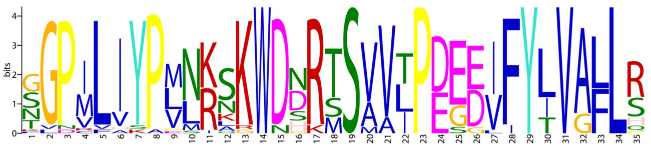

**motif 6**

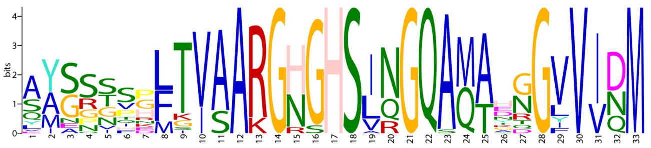

**motif 7**

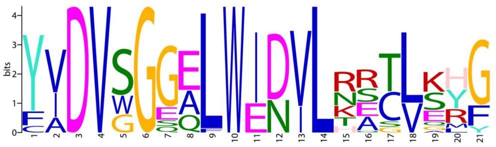

**motif 8**

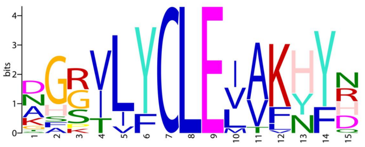

**motif 9**

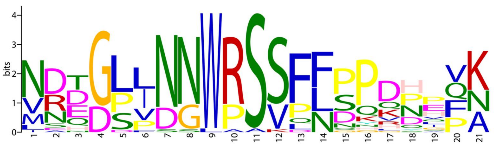

**motif 10**

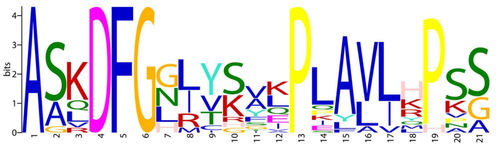

**Fig S2. Statistics of *cis*-acting elements**

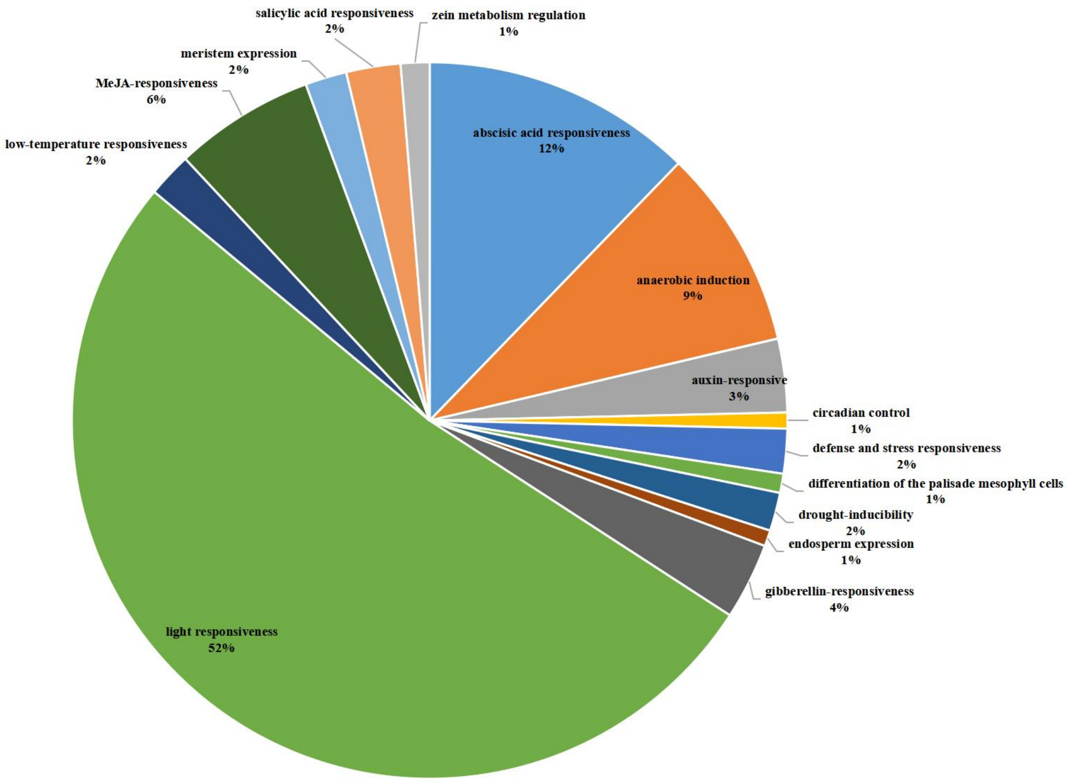

Supplement: Supplementary file 2 — Supplementary Material 2 [file 12870_2023_4419_MOESM2_ESM.pdf]
